# lncRNA RP11-10A14.5: a potential prognosis biomarker for LUAD through regulation on proliferation and metastasis

**DOI:** 10.1007/s12672-022-00493-2

**Published:** 2022-05-16

**Authors:** Zhijie Lin, Fenglan Feng, Jiaming Liang, Haikang Zeng, Jin Li

**Affiliations:** 1 The First Affiliated Hospital of Guangzhou Medical University/State Key Laboratory of Respiratory Diseases, Guangzhou Medical University, Guangzhou, 510120 China; 2 Department of Immunology, Institute of Translational Medicine, Medical College, Yangzhou University, Yangzhou, 225009 China

**Keywords:** Metastasis, Lung adenocarcinoma, Prognosis, Biomarker, RP11-10A14.5

## Abstract

Lung cancer is the malignancy most commonly seen worldwide. Emerging evidences indicated that lncRNAs may serve as a prognosis marker and play important role in NSCLC tumor biology. In this work, we analyzed the prognosis value of RP11-10A14.5 using TCGA and GEPIA database and expression profiles using PCR and FISH assay. The biological roles of RP11-10A14.5 in cell growth and invasion were determined by in vitro and in vivo experiments. Expression of RP11-10A14.5 is correlated with increased clinical stage and poor survival prognosis. In vitro experiments revealed that RP11-10A14.5 was widely expressed in lung cancer cell lines and mainly distributed in the cytoplasm and enhanced the growth, invasion and migration ability of NSCLC cell lines. Immunofluorescence assay suggested that RP11-10A14.5 may promote EMT by downregulating E-cadherin and upregulating N-cadherin and Vimentin. Flow cytometry results suggested that RP11-10A14.5 did not significantly affect cell cycle function, but could significantly inhibit apoptosis which may further enhance metastasis cell survival. In conclusion, RP11-10A14.5 is associated with clinical stage and poor survival outcome, may serve as a diagnosis and prognosis predictor for LUAD. Further, RP11-10A14.5 could promote LUAD cell growth and metastasis.

**Supplementary Information:**

The online version contains supplementary material available at 10.1007/s12672-022-00493-2.

## Introduction

Lung cancer is one of the most common type of cancers in the world [1]. Globally, 2.1 million new lung cancer cases were diagnosed each year, representing 11.6% of the total cases in 2018 [2]. In China, lung cancer is the first cause of death among all the malignancies since 2008 [3]. Lung cancer comprised of non-small cell lung cancer (NSCLC) and small cell lung cancer (SCLC). Non-small cell lung cancer is the most common type of lung cancer, accounting for about 85%. Among non-small cell lung cancers, about 50% of them are lung adenocarcinoma [1]. Early stage lung cancer patients are not easily detected due to the lack of clinical manifestations, and some of them have already undergone local progression by the time they are diagnosed [4]. Most LUAD patients are already at an advanced stage at the time of diagnosis, with an overall 5-year survival rate of only about 5% [5]. The currently used diagnostic tools such as ctDNA, tissue biopsy samples for diagnosis at the early stage of the disease and predicting the survival are inhibited by several limitations such as lack of specificity [6]. Therefore, identification of new biomarkers for diagnosis and prognosis of LUAD and exploring the underlying mechanism of development of LUAD is urgently needed for improving prognosis and treatment.

Long non-coding RNAs (lncRNAs) are a class of functional RNA molecules with transcripts longer than 200 nt (nucleotide units), which cannot encode proteins [7]. Many evidences indicated that dysregulated expression of lncRNAs are involved in the development and progression of tumors [8–10]. Emerging studies had revealed that many lncRNAs were aberrantly expressed in non-small cell lung cancer and found to be associated with tumor progression and survival outcome [11–14], suggesting that lncRNAs may play important role in lung cancer and could predict the survival prognosis of NSCLC patient.

In this study, to identify a potential prognosis biomarker for LUAD, we first identified differentially expressed lncRNAs in LUAD using TCGA and our LUAD cohort. Among these lncRNAs, the expression of FAM83A-AS1 and RP11-10A14.5 was the top 2 lncRNAs significantly positively correlated with clinical stage and associated with poor survival outcomes. FAM83A-AS1 has been reported in previously study that aggravated the malignant development of esophageal cancer by binding to miR-495-3p [15]. The role of RP11-10A14.5 remains unknown. In the present study, we investigated RP11-10A14.5’s potential role in the progression and development of LUAD.

## Materials and methods

### Cell culture

Human lung cancer cell lines (H1299, H1975, H441, A549, PC-9, H460, H1395, H1650, H358, H520, SPCA-1 and H1650) were cultured with RPMI-1640 or DMEM medium containing 10% fetal bovine serum (Gibco) and 1% penicillin–streptomycin (Gibco). All the cell lines were incubated in 37 °C incubator with 5% CO_2_ concentration.

### Human sample

Human lung cancer and paracancerous tissues were obtained from The First Affiliated Hospital of Guangzhou Medical University. The protocols used in human sample were conducted in according to the Ethical Review Committees of The First Affiliated Hospital of Guangzhou Medical University (2018-82). The written informed consent was provided from each individual.

### Plasmid construction and siRNA design

For RP11-10A14.5 overexpression, the full-length of RP11-10A14.5 was amplified by Phanta Master Mix (Vazyme, China) using primer (RP11-10A14.5-Forward: GATTCTAGAGCTAGCGAATTCATTGCGCATGCGGGAAAC, RP11-10A14.5-Reverse: ATCGCAGATCCTTCGCGGCCGCTTTTGCAGTGGGAGAAAAGAGG), and then inserted into the vector pCDH-CMV-MCS-EF1-CopGFP. The siRNA sequence targeting RP11-10A14.5 was designed by BLOCK-iT™ RNAi Designer (https://rnaidesigner.thermofisher.com/rnaiexpress/), an online siRNA design website provided by Thermo. The designed siRNA sequence was synthesized by GenePharma (Shanghai, China).

### RNA extraction and reverse transcriptase quantitative real-time PCR (RT-qPCR)

Total RNA was extracted using TRIzol reagent (Invitrogen) and the RNA concertation was determined by NanoDrop 2000. Then RNA was reverse transcribed to cDNA using a PrimeScript™ RT reagent kit (Takara, Japan) according to manufacturer’s instructions. RP11-10A14.5 expression was determined by SYBR Green Master Mix (Promega, Madison, WI, USA) on Biorad (Applied Biosystems Foster City, CA, USA). 2^−△△Ct^ method was used to calculate the relative expression of RP11-10A14.5. Primers for microRNA quantification were designed by miRAN Design software (Vazyme Biotech, Nanjing, China). microRNA expression was determined by miRNA 1st Strand cDNA Synthesis Kit by stem-loop and miRNA Universal SYBR qPCR Master Mix (Vazyme Biotech). All primer used in this study were showed in Table 1.

### RNA-fluorescence in situ hybridization (FISH)

The cellular distribution of RP11-10A14.5 was detected using FISH assay. H1299 cells were fixed using 4% formaldehyde and then hybridized with probe labeled with digoxigenin (Roche, cat #11277073910), and incubated at 37 °C overnight. Subsequently, cells were incubated with anti-digoxigenin-AP (Roche, cat #11363514910) for 30 min then incubated with diluted CSPD solution overnight and imaged using a microscope.

### Flow cytometric assay

H1299 cells were harvested 48 h after transfection by trypsinization and supernatant was removed. Then, cells were stained with Annexin V-APC and 7-AAD by using the AnnexinV-APC7-AAD apoptosis kit (Multi Science, A00933) following the manufacturer’s instruction then analyzed by flow cytometry (FACSverse; BD Biosciences). For cell cycle assay, H1299 cells were stained with PI by using the cell cycle and apoptosis analysis kit (Beyotime, China, C1052) following the manufacturer’s instruction, and the stained cells were analyzed using flow cytometry (FACSverse; BD Biosciences). The distribution of cells in G0/G1, S, and G2/M phase were calculated using FlowJo V10.

### Trans-well assay

Cell invasion assays was performed using a 24-well Transwell chamber (Corning, USA) coated with Matrigel (Corning). Cells (1 × 105) were added to the upper layer of chamber and 800 µl of 10% FBS medium was added to the lower layer of chamber. After 24 h of incubation, cells in the upper part of the chamber were wided away using a cotton swab. Cells that had invaded the lower chamber were fixed with 4% paraformaldehyde (PFA) for 30 min and stained with 1% crystal violet.

### MTT assay

Cells were seeded into the wells of 96-well plates (1000 cells/well) containing 100 ml of RPMI-1640 medium. Each well was added with 10 µl MTT solution, and the 96-well plate was incubated for 1 h at 37 °C. Then, the supernatants of each well were removed and 150 μl of dimethyl sulfoxide (DMSO) solution was added into each well and incubated for 15 min at 37 °C. Finally, the optical density (OD) value at 570 nm of each well was measured using microplate reader.

### Scratch assay

Cells were seeded into each well of the 24-well plate. A 200 μl pipette tip was used to scrape the cells and thereby creating a cell-free area among the cell monolayer. The change of the cell-free area was measured to evaluate the ability of the cell migration. The 24-well plate was incubated at 37 °C. Pictures were taken at 0, 12 and 24 h.

###  Fluorescence immunohistochemistry

Cells were fixed using 4% paraformaldehyde for 10 min at room temperature, then a permeabilization protocol was applied. The slides were then incubated with primary antibodies of E-Cadherin (BD, 610182), N-Cadherin (Cell Signaling, #13116) and Vimentin (Cell Signaling, #5741). After incubating with second antibodies of AF594-labeled anti-Mouse IgG (1:200) or AF647-labeled anti-Rabbit IgG (1:200), slides were incubated with DAPI (1:1000) for nuclear staining. The slides were imaged using a microscopy.

### Western blot

Cell lysis was attained using RIPA buffer (Beyotime, China, P0013) containing protease and phosphatase inhibitors (Beyotime, China, ST506) then concentration was determined using standard bicinchoninic acid (BCA) test (Beyotime, China, P0011) following manufacturer guideline. Proteins were separated on 12% SDS-PAGE gel and then transferred to PVDF membrane which was blocked using 5% nonfat milk, and incubated with primary antibodies overnight at 4 °C and then secondary antibody next day. The protein bands were detected using an ECL kit (Beyotime, China). The antibodies used were as follow: BAX (Affinity, AF0120), BAK (Affinity, AF0119), CASPASE-3 (Santacruz, SC-56053), GAPDH (Trevigen, 2275-PC-100), goat anti-rabbit IgG (Santacruz, sc2004).

### Identification of differentially expressed genes

The “limma” data package in R software was used to determine the differentially expressed gene between high and low RP11-10A14.5 expression groups [16]. An adjusted P-value < 0.05 and |logFC|> 0.5 were set as the cutoff criteria to identify the significantly differential expressed genes.

### Enrichment analysis

Gene set enrichment analysis (GSEA) was performed using the R package “Cluster Profiler” [17] to identify the enriched functional pathway in RP11-10A14.5 high and low expression group. We set the q value < 0.05 to prevent high false discovery rate. The adjusted P-value < 0.05 was set as the cutoff value of significant level.

### Predicting the mechanism by which RP11-10A14.5 regulates apoptosis protein expression

We predicted the target miRNAs of RP11-10A14.5 and the possible miRNAs bound to the target proteins using online database miRcode (http://www.mircode.org/). We used the animal transcription factor database (http://bioinfo.life.hust.edu.cn/AnimalTFDB/#!/tfbs_predict) predicting the possible binding transcription factors of RP11-10A14.5, as well as the transcription factors that may regulate the target protein.

### Survival analysis

Survival analysis for DEGs in LUAD was performed using GEPIA online database (http://gepia.cancer-pku.cn/) [18]. We divided the samples into high and low expression group based on the median expression. Cox regression analysis were performed to evaluate the association between the gene expression and survival prognosis.

### Mouse tumor models

H1299 cells (Control or RP11-10A14.5OV, 1 × 106 cells) were subcutaneously transplanted into the backs of 8–10 weeks female nude mice for 21 days. At 7 days after inoculation with WT H1299 cells, RP11-10A14.5 siRNA or scramble siRNA (5 nmol/kg) were intratumorally injected every 3 days for addition 2 weeks. Tumor growth was measured using calipers, and the tumor volume was calculated as V = (width)2 × length/2. All mice were sacrificed at day 22 for the collection of their lung and tumors.

### Statistical analysis

R (4.0.2) software was used to perform all statistical analyses. A two-tailed student t-test was used for comparison between two groups. Univariate Cox regression analysis was used to evaluate to analyze the association between clinicopathologic parameters and survival time. A two-sided P < 0.05 was considered statistically significant in all the statistical analysis.

## Results

### Expression profile and prognosis value of RP11-10A14.5 in LUAD

**Fig. 1:**
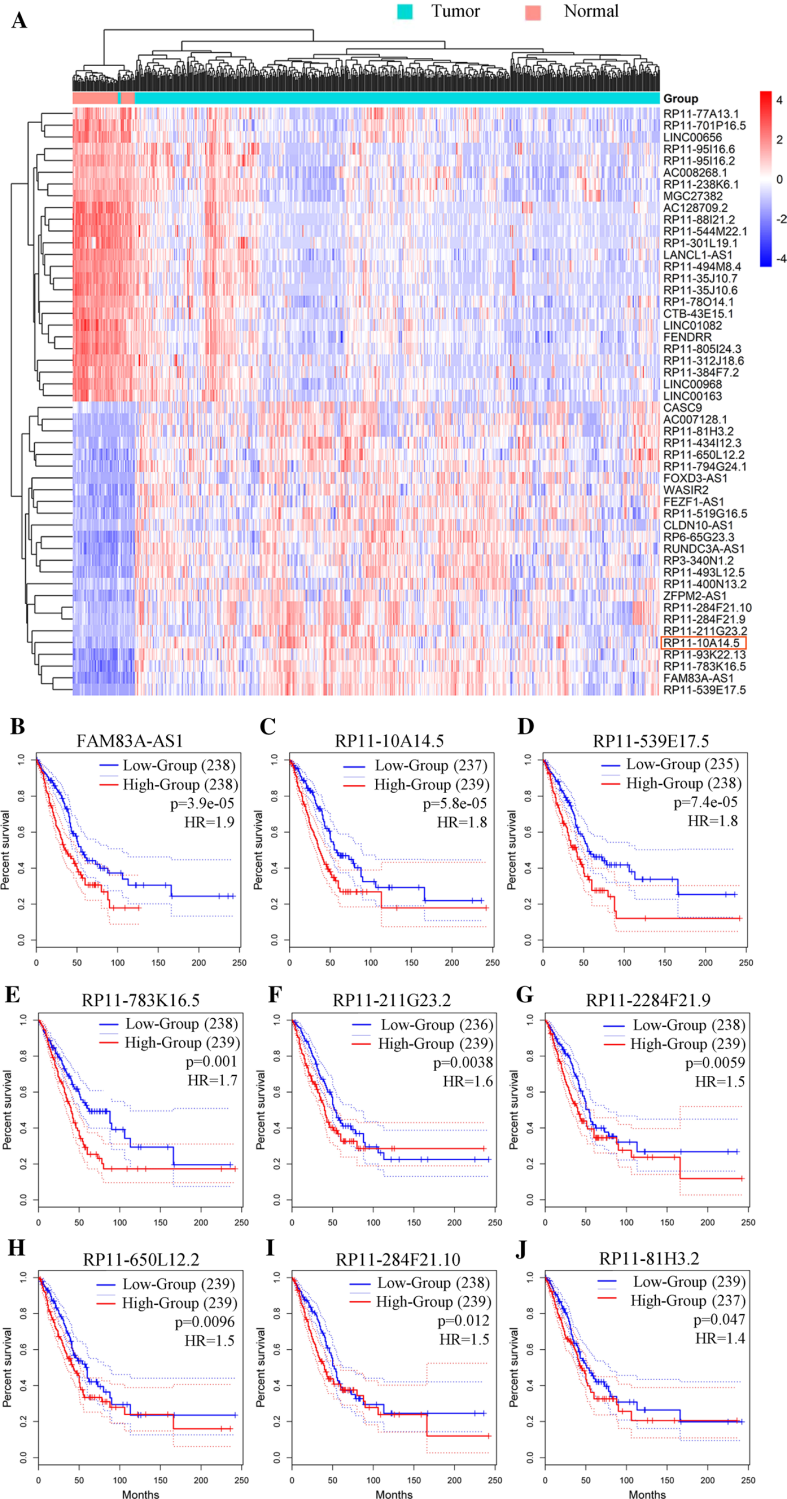
Differentially expressed lncRNAs in LUAD. **A** Heatmap showing the top 25 differentially up regulated and down regulated lncRNA in TCGA LUAD cohort. **B**–**J** Survival analysis for the top 9 differentially up regulated lncRNAs.

**Fig. 2:**
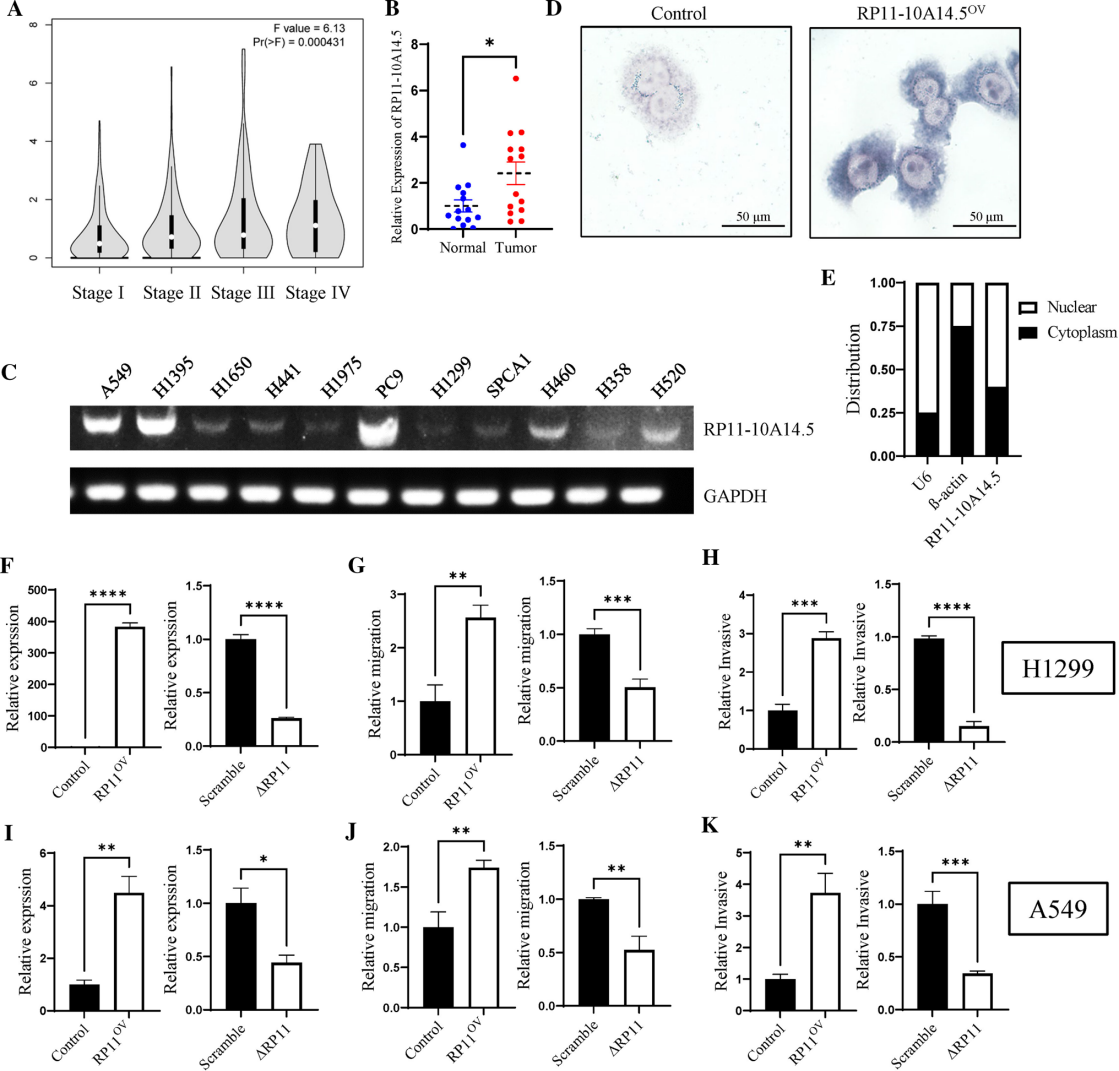
Expression and biological effects of RP11-10A14.5 on H1299. **A** Expression of RP11-10A14.5 in different stages of LUAD. **B** Expression of RP11-10A14.5 in lung adenocarcinoma was verified in lung cancer tissues. **C** Expression of RP11-10A14.5 in lung cancer cell was detected by PCR. **D** Cellular distribution of RP11-10A14.5 in H1299 was detected by FISH assay. Left panel represents the control group (H1299), right panel represents the overexpression group (H1299-RP11-10A14.5OV). **E** Cellular distribution of RP11-10A14.5 in H1299 cell line. **F** Expression of RP11-10A14.5 in H1299 stably expressed RP11-10A14.5 plasmid (RP11OV) and H1299 transfected with siRNA targeting RP11-10A14.5 (∆RP11). **G** Scratch assay assessing the migration ability of H1299-RP11OV and H1299-∆RP11 cells. **H** Trans well assay assessing the invasion ability of H1299-RP11OV and H1299-∆RP11 cells. **I** Expression of RP11-10A14.5 in A549 stably expressed RP11-10A14.5 plasmid (RP11OV) and A549 transfected with siRNA targeting RP11-10A14.5 (∆RP11). **J** Scratch assay assessing the migration ability of A549-RP11OV and A549-∆RP11 cells. **K** Transwell assay assessing the invasion ability of A549-RP11OV and A549-∆RP11 cells

We first identified differentially expressed lncRNA between tumor tissues and normal tissues using the TCGA LUAD cohort. Based on the cutoff criteria of |logFC|> 2 and adjusted P value < 0.05, we acquired a total of 175 differentially expressed lncRNAs of LUAD, 101 of which were differentially up-regulated lncRNAs and 74 were differentially down-regulated lncRNAs (Fig. 1A). We then analyzed the prognosis value of these lncRNAs. Among the top 25 differentially upregulated lncRNAs, RP11-10A14.5 out of nine (Fig. 1B–J) was significantly associated with survival prognosis ranked by P-value (Fig. 1C). Furthermore, we found that the high expression of RP11-10A14.5 in lung adenocarcinoma was significantly correlated with increased clinical stage (Fig. 2A, P < 0.001). High expression of RP11-10A14.5 in lung adenocarcinoma was verified in lung cancer tissues (Fig. 2B). Next, to investigate the expression and distribution of RP11-10A14.5 in LUAD, we first used PCR assay to detect the expression profile of RP11-10A14.5 in normal lung bronchial epithelium as well as lung cancer cell line. The results showed that RP11-10A14.5 was widely expressed in lung cancer cell lines (Fig. 2C). RP11-10A14.5 was weakly expressed in H1650, H441, H1975, H1299, SPCA1 and H358 cell lines, while it was significantly expressed in A549, H1395, PC9, H460 and H520 cell lines. FISH assay was performed to show that RP11-10A14.5 was mainly distributed in the cytoplasm and partially distributed in the nucleus (Fig. 2D, E).

### The manipulation of RP11-10A14.5 level altered the LUAD cells malignant behavior

We further explore the biological effects of RP11-10A14.5 on the LUAD cell line. LUAD cell line H1299 stably expressing RP11-10A14.5 (H1299-RP11OV) was constructed. The expression levels of RP11-10A14.5 in the H1299-RP11OV cell line were significantly upregulated compared to the control group, which was confirmed by RT-qPCR (Fig. 2F). Result of the scratch assay indicated that H1299 overexpressed of RP11-10A14.5 showed a significant enhance of cell motility (Fig. 2G), and the result of the transwell assay indicated that elevated expression of RP11-10A14.5 enhanced the invasion ability of LUAD cells (Fig. 2H). Next, we knockdown the expression level of RP11-10A14.5 in H1299 (H1299-∆RP11). The efficiency of knock down of RP11-10A14.5 was determined by RT-qPCR, showing that the expression level of RP11-10A14.5 of H1299 cells transduced with siRNA was about 75% lower than that of control cells (Fig. 2F). Reduced expression of RP11-10A14.5 significantly inhibited the migration and invasion ability of H1299 (Fig. 2 G, H). These results were also performed in A549 cells, and showed consistent results we obtained from H1299 cells (Fig. 2I–K).

### Effect of RP11-10A14.5 on the tumor growth and LUAD metastasis in vivo

**Fig. 3:**
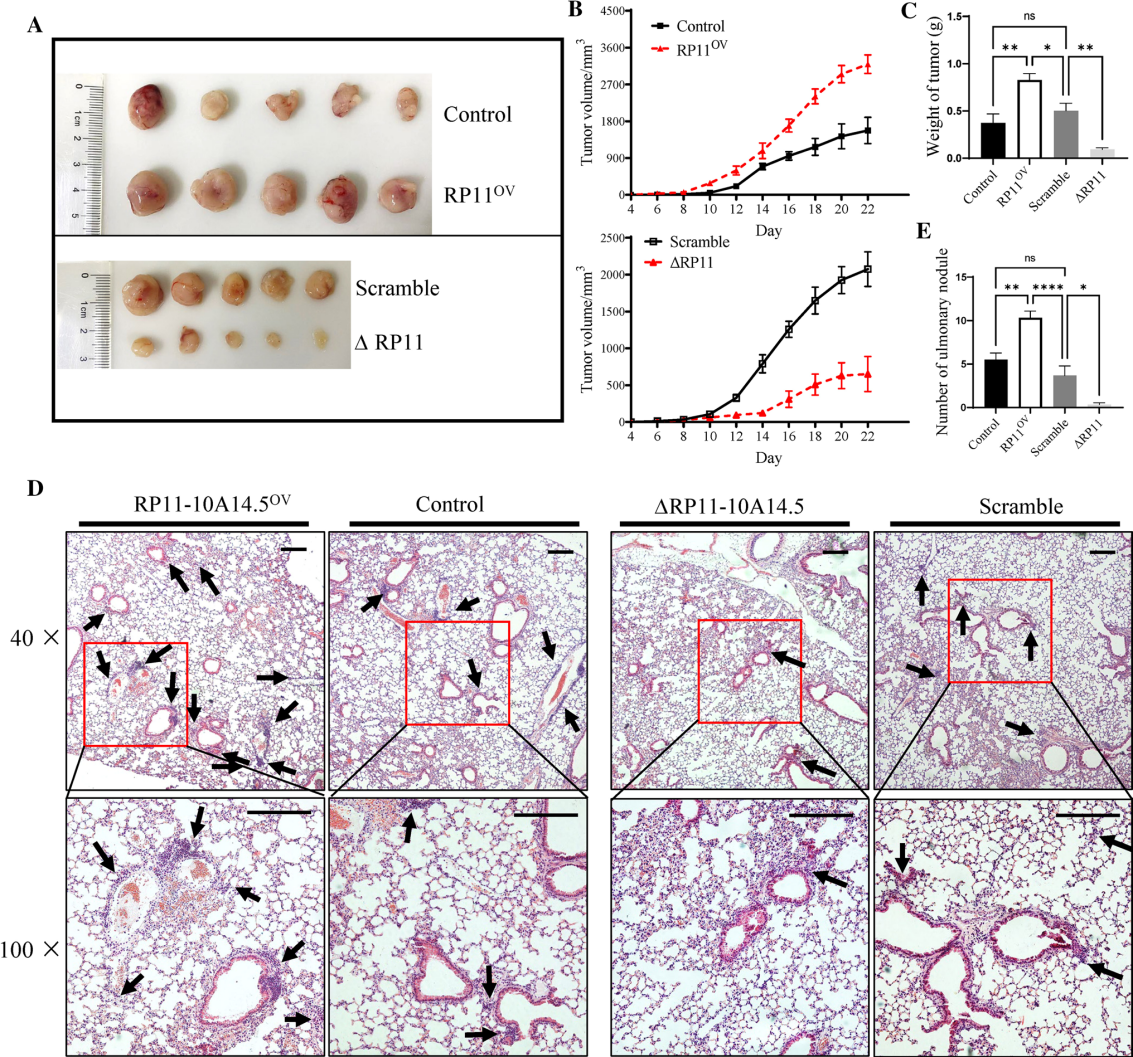
Effect of RP11-10A14.5 on the tumor growth and metastasis. **A** Tumor morphology of H1299-RP11OV and H1299-∆RP11 in tumor bearing mice. **B** Tumor growth of H1299-RP11OV and H1299-∆RP11 in vivo. **C** Tumor weight of H1299-RP11OV and H1299-∆RP11 in tumor bearing mice. Pulmonary nodule morphology (**D**) and numbers (**E**) in lung of tumor bearing mice

Next, we explore the effect of RP11-10A14.5 on the tumor growth and lung metastasis in nude mice. H1299-RP11OV or H1299-∆RP11 cells were subcutaneously transplanted into the backs of nude mice. Result of the tumor growth curve indicated that overexpress of RP11-10A14.5 promoted tumor growth, whereas knockdown of RP11-10A14.5 limited tumor growth in vivo (Fig. 3A, B). Tumor tissues were weighted. The results showed significant increased tumor weight in H1299-OV tumor bearing mice compared with the control or KD groups (Fig. 3C). Meanwhile, H&E staining of lung from tumor bearing mice indicated that overexpression of RP11-10A14.5 aggravated metastasis of LUAD cells, while knock down of RP11-10A14.5 inhibited tumor cell metastasis (Fig. 3D, E).

### Identification of differentially expressed genes and enrichment analysis

**Fig. 4:**
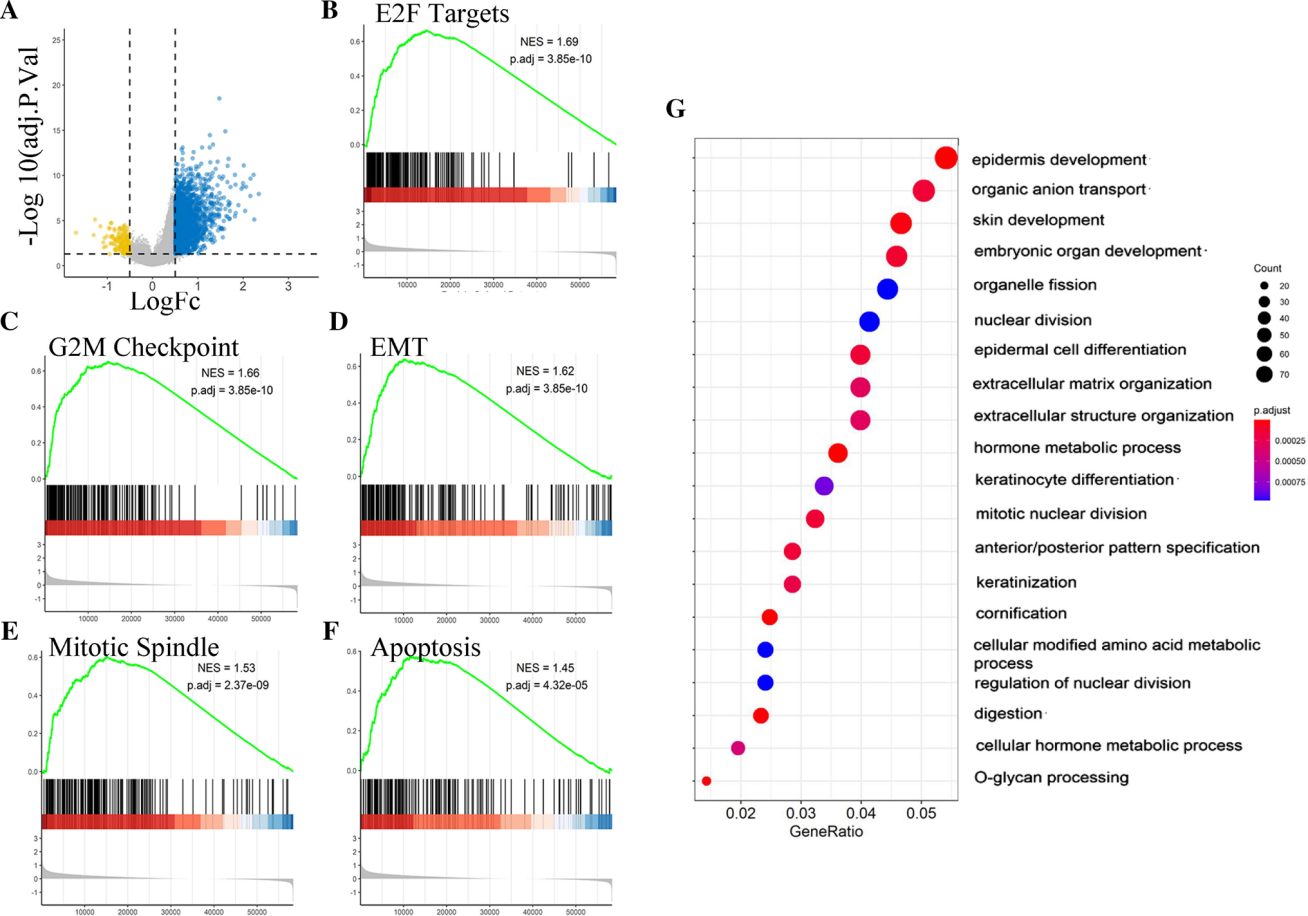
Differentially expression gene identification and the enrichment analysis of RP11-10A14.5-low and -high expression patients in TCGA LUAD cohort. **A** Volcano plot showed the differentially expressed genes. GSEA revealed the key pathways associated with RP11-10A14.5 regulation, including E2F targets (**B**), G2M checkpoint (**C**), Epithelial mesenchymal transition (**D**), Mitotic spindle (**E**) and Apoptosis (**F**). **G** GO enrichment analysis for up regulated genes associated with RP11-10A14.5 expression

A total of 2467 genes (including 2302 upregulated and 165 downregulated genes) were identified as DEGs between the high RP11-10A14.5 and low RP11-10A14.5 groups, as shown in the volcano plot (Fig. 4A). Next, GSEA was performed to identified biological pathway associated with RP11-10A14.5. The key pathways were identified, including “E2F target” (Fig. 4B, adjust P = 3.85e−10), “G2M checkpoint” (Fig. 4C, adjust P = 3.85e−10), “Epithelial Mesenchymal Transition” (Fig. 4D, adjust P = 3.85e−10), “Mitotic Spindle” (Fig. 4E, adjust P = 2.37e−9) and “apoptosis” (Fig. 4F, adjust P = 4.32e−10). Among those identified pathways, the biological pathways related to the invasion ability of tumor cells included “Epithelial Mesenchymal Transition”, and biological pathways related to cell growth including “E2F target”, “G2M checkpoint”, “Mitotic Spindle” and “apoptosis”. Our analysis results indicated that RP11.10A14.5 associated with cell cycle, apoptosis, and cell invasion-related biological pathways, suggesting that RP11-10A14.5 may promote the metastasis cell survival of lung adenocarcinoma through these biological pathways.

Next, GO enrichment analysis was performed for the upregulated DEGs to identify associated biological pathway. The attained results revealed that the upregulated DEGs were mainly enriched in cell growth-related biological pathways including “organelle division”, “nuclear division” and “mitotic nuclear division”, and biological pathways associated with cell invasion including “extracellular matrix organization” and “extracellular structure organization” (Fig. 4G). The above results suggest that RP11-10A14.5 may be associated with cell growth and cell invasion, and may promote the development of tumor through these biological pathways.

### RP11-10A14.5 promote EMT marker in LUAD cells

**Fig. 5:**
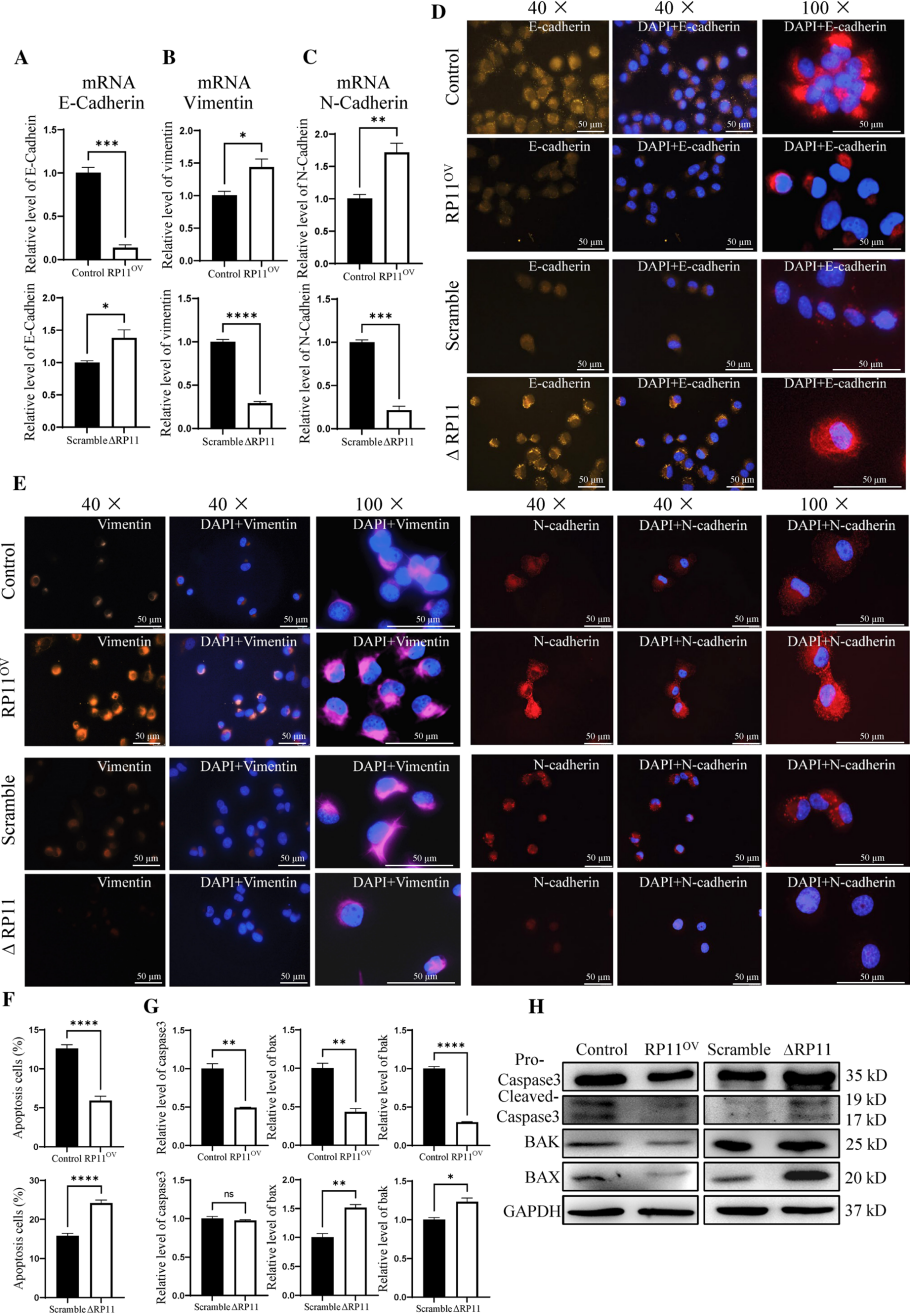
Effect of RP11-10A14.5 on the tumor metastasis markers and cell apoptosis. Relative expression of E-cadherin (**A**), Vimentin (**B**) and N-Cadherin (**C**) mRNA in H1299-RP11OV and H1299-∆RP11 cells. Expression of E-Cadherin (**D**), Vimentin (**E**) and N-Cadherin (**F**) in H1299-RP11OV and H1299-∆RP11 cells verified by IF. **F** Cell apoptosis rate of H1299-RP11OV and H1299-∆RP11 cells. **G** Relative expression of Caspase-3, Bax and Bak mRNA in H1299-RP11OV and H1299-∆RP11 cells. **H** Protein expression of Pro-Caspase-3, Cleaved-Caspase-3, Bak and Bax in H1299-RP11OV and H1299-∆RP11 cells.

Our previous experiments showed that RP11-10A14.5 could enhance the invasive ability of tumor cells, and our bioinformatical analysis suggested that RP11-10A14.5 was associated with apoptosis and invasion related pathways like EMT. To further investigate the mechanism of RP11-10A14.5 regulation of invasion as well as apoptosis, we first examined the effect of RP11-10A14.5 on the expression levels of EMT-related genes including E-cadherin, Vimentin and N-cadherin using QRT-PCR assay. The results showed that overexpression of RP11-10A14.5 downregulated the expression of E-cadherin, while upregulated the expression of Vimentin, and N-cadherin compared to the control group (Fig. 5A–C). Conversely, downregulation of RP11-10-14A.5 in H1299 cells increased E-Cadherin expression and decreased the expression of vimentin and N-cadherin (Fig. 5A–C). These data were further confirmed by immunofluorescence assay, as shown in Fig. 5D, E.

Next, we investigated whether RP11-10A14.5 could promote lung cancer cell growth by regulating cell apoptosis. The attained results suggested that overexpression of RP11-10A14.5 in H1299 significantly reduced the apoptosis level compared with the control group (Fig. 5F). While the apoptosis levels in H1299 RP11-10A14.5 knockdown cells were significantly higher than control (Fig. 5F). The expression levels of apoptotic proteins including BAK, BAX and Caspase-3 were reduced in cells overexpressed RP11-10A14.5 compared to the control group (Fig. 5G, H); the expression levels of apoptotic proteins including BAK, BAX, and Caspase-3 were increased in RP11-10A14.5 knockdown NSCLC cells, compared to the control group (Fig. 5G, H).

### The construction of functional model in LUAD by lncRNA RP11-10A14.5

**Fig. 6:**
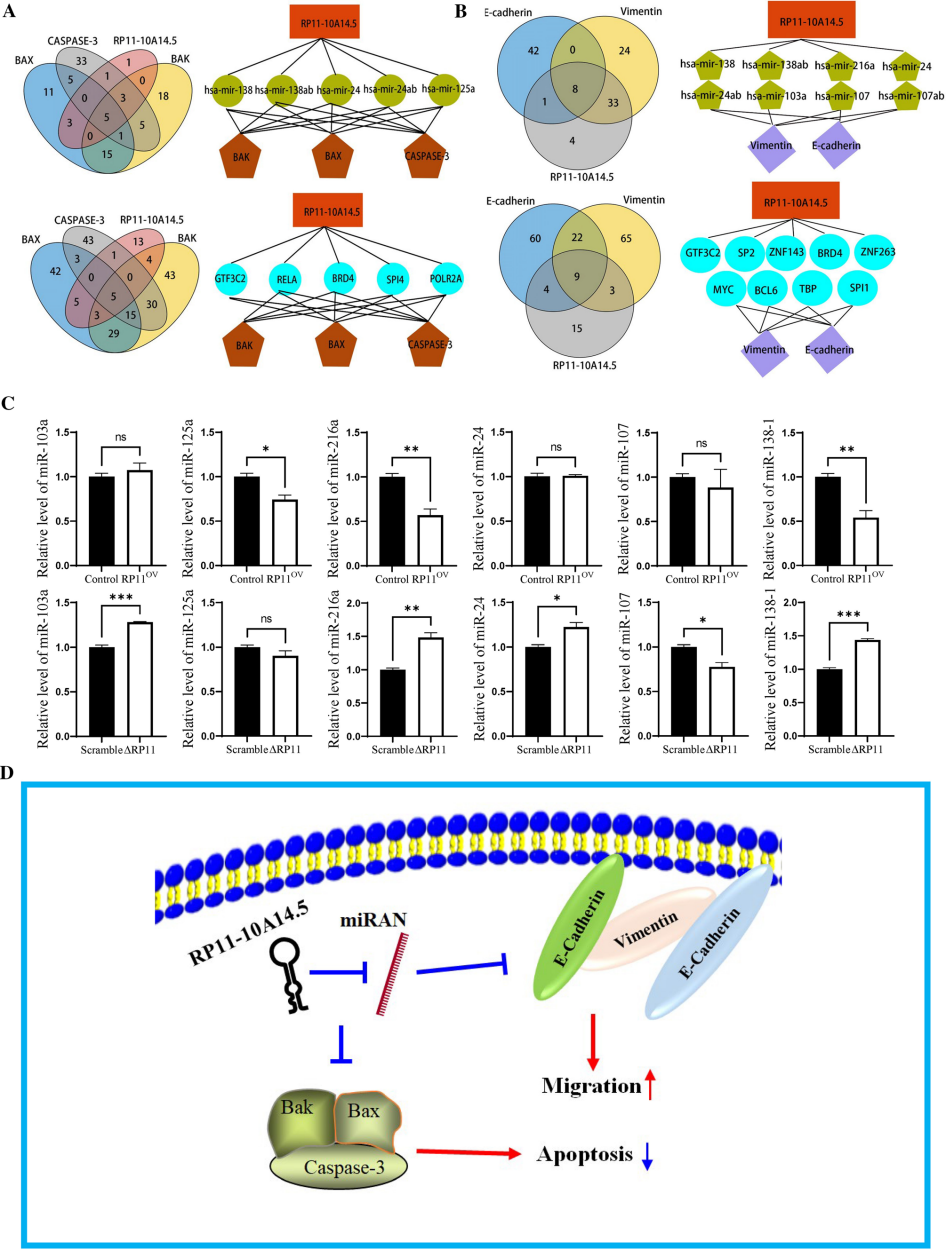
**A** Venn diagram showing the predicted miRNAs and transcription factors associated with RP11-10A14.5 to regulate apoptotic genes. **B** Venn diagram showing the predicted miRNAs and transcription factors associated with RP11-10A14.5 to regulating metastasis markers. **C** QRT-PCR verify the predicted miRNA in H1299-RP11OV and H1299-∆RP11cells. **D** Working model of RP11-10A14.5 regulates apoptotic and metastasis in LUAD cells

To explore the possible mechanisms by which RP11-10A14.5 regulates the expression of EMT markers and apoptotic proteins, we first predicted the miRNAs bound by RP11-10A14.5 and target proteins using online database miRcode. There are 15, 48, 66, 78, 183 and 91 miRNAs are predicted to bind on RP11-10A14.5, BAX, BAK, Caspase-3, E-cadherin and Vimentin respectively (Fig. 6A, B). We attained 5 and 8 miRNAs by which RP11-10A14.5 predicted to regulate those genes. It is suggested that RP11-10A14.5 may regulate the expression of BAK, BAX, and CASPASE-3 through hsa-mir-138, hsa-mir-138ab, hsa-mir-24, hsa-mir-24ab, and hsa-mir-125a (Fig. 6A), and the expression of E-cadherin and Vimentin through has-mir-138, has-mir-138ab, has-mir-216a, has-mir-24, has-mir-24ab, has-mir-103a, has-mir-107 and has-mir-107ab (Fig. 6B). In addition, we predicted RP11-10A14.5 and the possible transcription factors bound by the target genes from the animal transcription factor database. There are 31, 102, 129, 97, 95 and 99 transcription factors that predicted to bind on RP11-10A14.5, BAX, BAK, Caspase-3, E-cadherin and Vimentin respectively (Fig. 6A, B). We attained 5 and 9 transcription factors by which RP11-10A14.5 predicted to regulate those genes. It is suggested that RP11-10A14.5 may regulate the expression of BAK, BAX, and Caspase-3 through transcription factors including GTF3C2, RELA, BRD4, SPI1, and POLR2A (Fig. 6A), and the expression of E-cadherin and Vimentin by GTF3C2, SP2, ZNF143, BRD4, ZNF263, MYC, BCL6, TBP and SP1 (Fig. 6B). Next, we verified the association of expression levels between predicted miRNAs and RP11-10A14.5 in overexpression and knockdown H1299 cell line (Fig. 6C).

## Discussion

Emerging studies suggest that lncRNAs are aberrantly expressed in LUAD and may play important role in the development of lung cancer, and correlate with clinical stage and survival outcomes [19–21]. Furthermore, lncRNAs are widespread in different kinds of body fluids and show potential for clinical diagnosis and prognosis for LUAD patient [10]. In this current study, to identify lncRNAs associated with prognosis outcomes with LUAD, we first identified differentially expressed lncRNAs in LUAD using the TCGA LUAD cohort, and explored the prognosis value of these lncRNAs. Among them, FAM83A-AS1 and RP11-10A14.5 was most significantly associated with the survival prognosis of patients. The high expression of RP11-10A14.5 is correlate with the clinical progression of LUAD and promote tumour cell metastasis in vitro and in vivo. RP11-10A14.5 was widely expressed in lung cancer cell lines, and mainly distributed in cytoplasm, suggesting that RP11-10A14.5 may exerted its biological function in the cytoplasm.

Next, we further explored the underlying biological mechanism of RP11-10A14.5 in LUAD. GSEA and GO enrichment analysis showed that RP11-10A14.5 is associated with invasion-related biological pathways such as epithelial mesenchymal transition [22], extracellular matrix organization. In this study, RP11-10A14.5 shows the ability to induce the expression of EMT markers in tumor cells which may contribute to the process of EMT, suggesting its association with the aggressiveness of tumors. Moreover, our bioinformatical analysis also revealed that RP11-10A14.5 expression was related to cell proliferation related biological pathways as apoptosis. Our in vitro results suggest that RP11-10A14.5 might inhibit tumor cell apoptosis through the regulation on expression of BAK, BAX and Caspase-3, which might enhance the metastatic cell survival.

We further constructed the RNA network and verified the target miRNA by RT-PCR miRNAs-138, -24, -125, -216, -103, 107 and transcription factors GTF3C2, RELA, BRD4, SPI4, PCLR2A, SP2, ZNF143, ZNF263, MYC, BCL6, TBP, SPI1 were predicted associates with RP11-10A14.5 to regulating apoptotic and EMT genes. Expression of miR-216a and miR-138-1 were significant decreased in RP11-11A14.5 overexpressed H1299 cells, and in contrarily, these miRNAs were upregulated in RP11-10A14.5 knockdown H1299 cells. Further study may focus on interaction between miR-216a, miR-138-1 and RP11-10A14.5 to investigate the potential biological mechanism of RP11-10A14.5 in LUAD. Together, we provided the hypothesis: RP11-10A14.5 may promote the proliferation of LUAD and the invasion of tumor cells through the predicted miRNAs and transcription factors to inhibit cell apoptosis and EMT related genes in LUAD (Fig. 6D).

## Conclusion

The expression of RP11-10A14.5 is associated with clinical stage and poor survival outcome, indicating that RP11-10A14.5 may serve as a diagnosis and prognosis biomarker for LUAD. Further, in vitro and in vivo experiment showed that RP11-10A14.5 could promote LUAD cell growth and metastasis.

## Supplementary Information

Additional file 1 (PDF 162 KB)

## Data Availability

The data presented in this study are available on request from the corresponding author.
