# Supplementary material for: lncRNA RP11-10A14.5: a potential prognosis biomarker for LUAD through regulation on proliferation and metastasis
Source: Discov Oncol. 2022 May 16;13:32. doi: 10.1007/s12672-022-00493-2 (PMC9110618; doi:10.1007/s12672-022-00493-2)
Supplement: Supplementary file 1 — Additional file 1 (PDF 162 KB) [file 12672_2022_493_MOESM1_ESM.pdf]

Table S1 Primers used in this study

Primer name	Primer sequences 5`-3`
RT-miR-24	GTCGTATCCAGTGCAGGGTCCGAGGTATTCGCACTGGATA CGACCTGTTC
miR-24-F	GCGTGGCTCAGTTCAGCAG
RT-miR-103a	GTCGTATCCAGTGCAGGGTCCGAGGTATTCGCACTGGATA CGACCGGCGC
miR-103a-F	CGCGCGCGGCCACAG
RT-miR-107	GTCGTATCCAGTGCAGGGTCCGAGGTATTCGCACTGGATA CGACTGTGCC
miR-107-F	GCGCGAGCAGCAGACAG
RT-miR-125a	GTCGTATCCAGTGCAGGGTCCGAGGTATTCGCACTGGATA CGACGGCTCC
miR-125a-F	CGCGACAGGTGAGGTTCTTG
RT-miR-138	GTCGTATCCAGTGCAGGGTCCGAGGTATTCGCACTGGATA CGACCGGCCT
miR-138-F	GCGAGCTGGTGTTGTGAATC
RT-miR-216	GTCGTATCCAGTGCAGGGTCCGAGGTATTCGCACTGGATA CGACTCACAG
miR-216-F	CGCGTAATCTCAGCTGGCAA
BAK-F	CCATCAGCAGGAACAGGAGG
BAK-R	GGTGGCAATCTTGGTGAAGTA
BAX-F	TTTTGCTTCAGGGTTTCATCCA
BAX-R	GGGCCTTGAGCACCAGTTTG
Cas3-F	TGCTATTGTGAGGCGGTTGT
Cas3-R	GAATGTTTCCCTGAGGTTTGC
E-Cadherin-F	GAAGGAAGAGCTGCGATTTA
E-Cadherin-R	TGTGAAGGTGATTTCGGTGT
N-Cadherin-F	GTGGCGGAGATCCTACTGGAC
N-Cadherin-R	CCCTTGGCTAATGGCACTTGA
RP11-10A14.5-F	CGATTTCCTGACCTCGTCATCC
RP11-10A14.5-R	AGAAGCAACTTCAGTCCTTGCC
GAPDH-F	TGCACCACCAACTGCTTAGC
GAPDH-R	GGCATGGACTGTGGTCATGAG
